# Merits and Limitations of Studying Neuronal Depolarization-Dependent Processes Using Elevated External Potassium

**DOI:** 10.1177/1759091420974807

**Published:** 2020-11-30

**Authors:** Kira D. A. Rienecker, Robert G. Poston, Ramendra N. Saha

**Affiliations:** Department of Molecular and Cell Biology, School of Natural Sciences, University of California, Merced, United States

**Keywords:** L-type voltage sensitive calcium channels, immediate early genes (IEG), transcription, intracellular calcium, extracellular potassium

## Abstract

Elevated extracellular potassium chloride is widely used to achieve membrane depolarization of cultured neurons. This technique has illuminated mechanisms of calcium influx through L-type voltage sensitive calcium channels, activity-regulated signaling, downstream transcriptional events, and many other intracellular responses to depolarization. However, there is enormous variability in these treatments, including durations from seconds to days and concentrations from 3mM to 150 mM KCl. Differential effects of these variable protocols on neuronal activity and transcriptional programs are underexplored. Furthermore, potassium chloride treatments *in vitro* are criticized for being poor representatives of *in vivo* phenomena and are questioned for their effects on cell viability. In this review, we discuss the intracellular consequences of elevated extracellular potassium chloride treatment *in vitro*, the variability of such treatments in the literature, the strengths and limitations of this tool, and relevance of these studies to brain functions and dysfunctions.

## Introduction

Our understanding of the complex molecular mechanisms of brain development, physiology, and disorder are greatly aided by simple model systems, including dissociated neuronal cell culture. Here, immature neurons obtained from embryonic brains are cultured into mature neurons *in vitro*. Such dissociated cells are extensively used in neuroscience and are particularly useful in the study of neuronal excitation-transcription coupling. *In vitro*, elevated extracellular potassium is often employed to depolarize the neuronal membrane (Greenberg et al., 1985; Bartel et al., 1989; Gotoh et al., 1999; Michod et al., 2012) and induce Ca^2+^ influx through L-type voltage sensitive calcium channels (L-type VSCCs) (Ghosh et al., 1994; Xia et al., 1996; Tao et al., 1998; Martinowich et al., 2003; Greer and Greenberg, 2008; Malik et al., 2014; Qiu et al., 2016; Azad et al., 2018; Tyssowski et al., 2018), consequently initiating signaling cascades (Bading et al., 1993; Dolmetsch et al., 2001; Redmond et al., 2002; Kingsbury et al., 2007; Greer and Greenberg, 2008; Li et al., 2009; Lyons and West, 2011; Pruunsild et al., 2011; Evans et al., 2013; Cohen et al., 2018; Tyssowski et al., 2018) that result in activity responsive gene transcription (West et al., 2002; Martinowich et al., 2003; Greer and Greenberg, 2008; Kim et al., 2010; Pruunsild et al., 2011; Michod et al., 2012; Malik et al., 2014; Madabhushi et al., 2015; Azad et al., 2018; Tyssowski et al., 2018). In this review, we provide a general overview of these processes. We also discuss strengths and weaknesses of experimental approaches using elevated extracellular KCl (K^+^), their relevance to certain brain disorders, and offer suggestions regarding their possible use in other neurobiology subfields.

## Mechanisms at Play

### Elevated Extracellular KCl Induces Neuronal Depolarization, But Often, Not Activity

The neuronal membrane voltage is regulated by, among other factors, the concentration of potassium and sodium ions in the intracellular versus extracellular space. At rest, K^+^ is more concentrated inside the neuron, while Na^+^ concentration is higher outside the cell. Sodium-potassium pumps maintain these gradients, using ATP to pump Na^+^ out of the cell and K^+^ into the cell, against their gradients. Neurons at rest *in vivo* have a membrane voltage around -65 mV, whereas *in vitro*, they tend to be around -60 mV due to slightly higher KCl concentrations in commercial culture media formulations (5mM) in comparison to cerebrospinal fluid (3mM). Because the resting neuronal membrane is highly permeable to K^+^, the membrane potential is sensitive to changes in the extracellular potassium concentration – increasing extracellular potassium depolarizes neurons. As the extracellular concentration of potassium rises, the magnitude of the potassium gradient is reduced, and the potassium equilibrium potential becomes more positive. As external Na^+^ continues to leak into the soma, global depolarization occurs. As calculated by the Goldman equation, a tenfold change in extracellular K^+^ from 5 to 50mM will cause a 48mV membrane depolarization under physiological conditions. Experimentally, reminiscent of the Nernst relationship, elevation of external K^+^ to 20, 30, and 40mM concentration in one study depolarized the membrane to -37, -26, and -19mV respectively; the specific membrane potential achieved at 10 mM KCl was not reported in text, but was approximately -56mV in Figure 2B (Wheeler et al., 2008).

**Figure 1.**
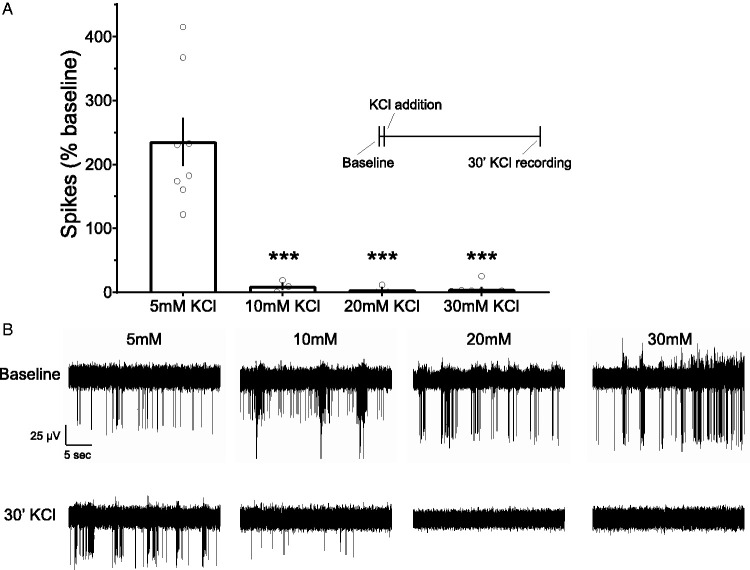
Neuronal Activity Under Elevated Extracellular KCl *In Vitro.* A: Spikes under KCl treatment as a % of baseline activity before treatment. Spiking is observed under 5mM KCl treatment, which matches [KCl] in standard media conditions, but disappears as [KCl]_o_ rises. Spike data were compared using ANOVA. *** *P* < 0.001. B: Example recordings during baseline measurements (top) and KCl treatments (bottom).

What happens to neuronal activity post membrane depolarization in response to elevated external potassium? When treated with 8mM KCl, spontaneous activity is retained in cortical neurons, but the pattern changes from burst firing to tonic spike firing (Golbs et al., 2011). However, application of 15mM KCl or higher, somewhat unintuitively, leads to a complete attenuation of spontaneous neuronal activity in dissociated hippocampal neurons (Grubb and Burrone, 2010). In our laboratory, we see similar activity arrest in dissociated cortical neurons, when treated with 10mM or higher concentrations of external K^+^ (Figure 1). Such obliteration of neuronal activity may be explained by depolarization-sensitive inactivation of voltage-gated channels. Alternatively, or in addition, mild depolarization could deplete pre-synaptic terminals of neurotransmitters, thereby preventing subsequent rounds of activity (Grubb and Burrone, 2010). Although 10mM or higher external K^+^-induced depolarization doesn’t produce neuronal activity, the sustained depolarization produces a chronic increase in an important second messenger, intracellular calcium (more in the next segment, 2.2) (Collins et al., 1991; Grubb and Burrone, 2010). Also, such sustained depolarization-coupled inactivation of neuronal activity may have notable functional repercussions in the brain (see segment 4.3.3).

### Calcium Influx

Both brief and prolonged stimulation with KCl elevate intracellular calcium in a sustained fashion for the duration of treatment (Dolmetsch et al., 2001; Kingsbury et al., 2007; Evans et al., 2013; Tyssowski et al., 2018). When the calcium chelator EGTA is included during KCl treatment, it blocks the increase in intracellular calcium, induction of *BDNF* mRNA, dendritic growth, and enhanced survival brought about by KCl (Zafra et al., 1990; Ghosh et al., 1994; Tao et al., 1998; Redmond et al., 2002; Tao-Cheng, 2018). In some cases, these changes are likewise prevented by reduction of the calcium concentration in the culture medium (Zafra et al., 1990). Together, these observations suggest that most of the biological effects of elevated external potassium are mediated by intracellular calcium signaling. Notably, in contrast to the localized depolarization that comes with synaptic stimulation, KCl administration induces global depolarization. Depolarization-induced calcium influx to the soma is possible through several different channels, including NMDA receptors, calcium permeable AMPA receptors, various voltage-sensitive calcium channels (VSCCs), and intracellular Ca^2+^ stores in the endoplasmic reticulum (Lyons and West, 2011). NMDA receptors are found at the synapse, but VSCCs are found globally across the plasma membrane–both at the synapse and at the soma. Studies with L-type Ca^2+^ channel antagonists like the dihydropyridines (DHPs) nimodipine and nifedipine show that L-type VSCCs mediate much of the effect of membrane depolarization by elevated extracellular KCl. Nimodipene is reported to attenuate KCl-induced Ca^2+^ influx by 15-20% (Dolmetsch et al., 2001) and nifedipine by 30-50% (Redmond et al., 2002). However, N- and P/Q-type channel blockers and NMDA receptor blockers can also reduce intracellular calcium concentrations induced by membrane depolarizations by 15-20% (Dolmetsch et al., 2001). Together, L-type VSCCs, N- and P/Q-type channels, and NMDARs each contribute to sustained intracellular calcium elevation in the cytoplasm and nucleus during potassium mediated depolarization (Dolmetsch et al., 2001; Lyons and West, 2011). It is notable that although Ca^2+^ influx is an absolute requirement for depolarization-induced downstream signaling, reduction of Ca^2+^ influx only marginally impairs signaling, suggesting the signaling strength is non-linearly coupled with intracellular calcium levels.

One of the key downstream mediators of calcium influx is the transcription factor CREB. CREB-dependent transcription signaled by Ca^2+^-CaMKII activation relies on the frequency of L-type channel openings, rather than bulk intracellular Ca^2+^ levels (Wheeler et al., 2008). Furthermore, N- and P/Q-type channel inhibitors (ω-Conotoxin GVIA and ω-Agatoxin IVA respectively), are unable to inhibit CREB activation and CREB-dependent transcription across various extracellular Ca^2+^ concentrations. In contrast, the L-type channel inhibitor nimodipine inhibits CREB Ser^133^ phosphorylation at concentrations of extracellular Ca^2+^ up to 20 mM (Dolmetsch et al., 2001). For these reasons, Ca^2+^ influx through the L-type channels is thought to be primarily responsible for Ca^2+^-dependent signaling and CREB-dependent transcription induced by elevated extracellular potassium. The majority of this review will focus on signaling from L-type VSCCs.

### L-Type VSCCs

Although multiple channels contribute to depolarization-coupled Ca^2+^ influx, L-type (long-lasting activation) channels provide specificity to the effects of elevated extracellular KCl in comparison to other stimulation paradigms (Ghosh et al., 1994; Lyons and West, 2011; Lyons et al., 2016). Induction of BDNF expression by KCl is completely blocked by L-type channel blockers nifedipine and nimodipine (Zafra et al., 1990; Ghosh et al., 1994; Lyons et al., 2016), but is not affected by the NMDAR blocker APV (D-2-amino-5-phosphonovaleric acid) or by CNQX (6-cyano-7-nitroquinoxaline-2,3-dione), an AMPAR/kainate (aka, non-NMDAR) blocker (Ghosh et al., 1994). In contrast, BDNF induced by glutamate is sensitive to APV, but not nifedipine or CNQX (Ghosh et al., 1994). Differential regulation and expression profiles of BDNF induced by signaling through NMDARs and VSCCs may explain the observation that calcium influx through NMDARs is more excitotoxic than calcium influx through L-type VSCCs (Ghosh et al., 1994). VSCCs sustain BDNF expression longer than NMDAR signaling. While calcium influx through either channel may be potentially excitotoxic, robust BDNF induction after VSCC activation may be an effective neuroprotectant, in contrast to the transient and weaker BNDF induction by NMDAR activation (Ghosh et al., 1994). More information about L-type VSCCs may be found in Lipscombe et al. (2004), Berger and Bartsch (2014), and Kobrinsky (2015).

### Signaling to the Nucleus

**Figure 2.**
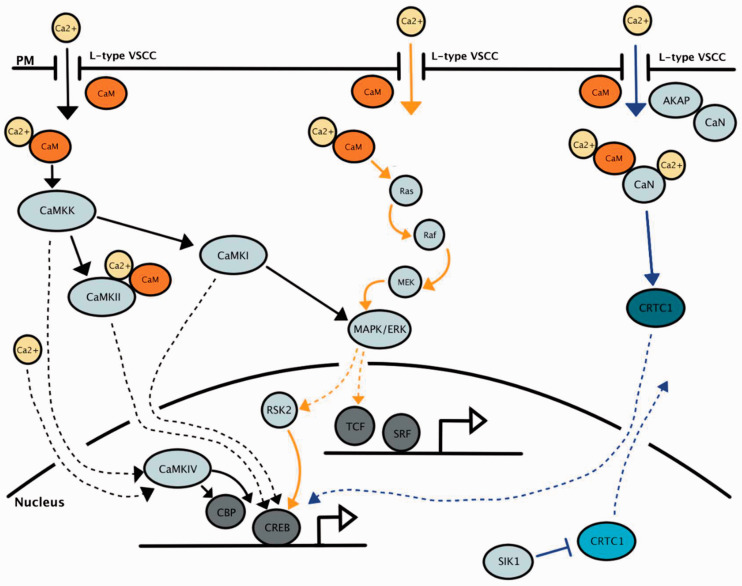
CaMK, MAPK/ERK, and CaN Signaling From L-VSCCs to Transcription. Signaling pathways initiating from Ca^2+^ influx at L-type VSCCs leading to transcription in the nucleus. Solid arrows represent intra-somatic interactions. Dashed arrows represent translocation into the nucleus. Black arrows represent the CaMK pathway, orange arrows represent the MAPK/ERK pathway, and blue arrows represent the CaN pathway. Dephosphorylated CRTC1 (in dark blue) translocates into the nucleus, and phosphorylated CRTC1 (in light blue) moves out of the nucleus.

Once Ca^2+^ influx occurs through L-type VSCCs, signaling cascades transmit this information to the nucleus. On certain occasions, information is postulated to be directly relayed by C-terminus fragments of L-type VSCCs (Gomez-Ospina et al., 2006). However, in many instances, the L-type channel activity is locally computed by Ca^2+^/CaM-dependent protein kinase II (CaMKII; see “Calmodulin Kinase (CaMKII/CaMKIV)” section), which then conveys the signal to downstream pathways. The principal cascades include the calmodulin kinase (CaMK) (Bading et al., 1993; Cohen et al., 2018), mitogen activated protein kinase (MAPK) (Ghosh and Greenberg, 1995; Tyssowski et al., 2018), and calcineurin pathways (Figure 2) (Rajadhyaksha and Kosofsky, 2005; Greer and Greenberg, 2008; Li et al., 2009), which converge on CREB-mediated transcription of activity-regulated genes. While all three pathways may be activated by calcium influx, high intracellular Ca^2+^ concentrations [Ca^2+^]_i_ preferentially promote kinase activation (CaMK and MAPK), while modest or low [Ca^2+^]_I_ preferentially activates the phosphatases (CaN) (Rajadhyaksha and Kosofsky, 2005). Additional recommended readings about signaling originating at L-type VSCCs include: Hagenston and Bading (2011), Greer and Greenberg (2008), and West et al. (2001).

#### Calmodulin Kinase (CaMKII/CaMKIV)

Calmodulin (CaM) binds the L-type VSCCs at the “IQ” motif in the carboxyl terminus of the channel (Dolmetsch et al., 2001). When extracellular KCl induces calcium influx through these channels, Ca^2+^ binds CaM (Ca^2+^/CaM), inducing a conformational switch that allows calmodulin to activate calmodulin kinase signaling. Ca^2+^/CaM activates cytosolic CaMKK, which in turn activates cytosolic CaMKI and CaMKII, or nuclear CaMKIV (Ghosh and Greenberg, 1995; Greer and Greenberg, 2008; Hagenston and Baing, 2011). CaMKII shuttles Ca^2+^/CaM to the nucleus and phosphorylates activity-dependent transcription factors including CREB, NeuroD, NF-kB, and MECP2 (Greer and Greenberg, 2008; Cohen et al., 2018). CaMKIV, activated by CaMKK and increases in nuclear [Ca^2+^], also activates CREB by phosphorylation at Ser 133 (Redmond et al., 2002). To successfully induce CREB-mediated transcription, CaMKIV must also activate the CREB co-activator CBP (CREB binding protein), a histone acetyltransferase (Hagenston and Baing, 2011). Kinase-dead CaMKIV, elimination of CaMKII, and dominant-negative CREB each block KCl-induced IEG transcription and dendritic growth (Redmond et al., 2002; Cohen et al., 2018). Both L-type VSCCs and NMDARs evoke CaMK signaling, which is critical for propagating the L-type VSCC signaling to the nucleus. However, distinctions between signaling from NMDARs and L-type VSCCs activate transcription at different regulatory elements of IEGs, indicating the mode of Ca^2+^ entry to the cell results in transcriptional specificity in the nucleus (Bading et al., 1993).

#### Ras/MAPK/ERK

CaM bound to the L-type VSCCs also activates Ras/MAPK/ERK signaling (Dolmetsch et al., 2001; Hagenston and Baing, 2011). Ca^2+^/CaM activates the GTP-binding protein Ras, which binds and activates Raf (mitogen activated protein kinase kinase), which phosphorylates and activates MEK. MEK phosphorylates and activates MAPK/ERK, which translocates to the nucleus to phosphorylate TCF-family transcription factors and stimulate TCF/SRF-dependent transcription (Hagenston and Baing, 2011). Ras/MAPK signaling also activates nuclear Rsk kinases, including Rsk2, which phosphorylates CREB at Ser-133 (West et al., 2001). Ca^2+^/CaM activates Ras/MAPK and CaMK pathways in concert, and both may lead to CREB-dependent transcription, but these signaling pathways may be responsive to different timing and magnitudes of neuronal activation (West et al., 2001; Hagenston and Baing, 2011). For instance, MAPK/ERK inhibition blunts and delays the first wave of IEG induction, whereas later waves of IEG transcription are less impacted, if at all (Tyssowski et al., 2018). Also, crosstalk exists between the CaMK and the MAPK pathways. Several groups, including ours, have shown that CaMKI regulates depolarization-induced MEK-ERK activity and their physiological functions (Schmitt et al., 2004, 2005; Wayman et al., 2006; Poston et al., 2020). We have recently identified that certain ortho-hydroxylated brominated ethers, which are persistent environmental toxins widely detectable in organisms including humans, bind to and inhibit CaMKI, which subsequently impairs MEK-ERK-dependent neuronal functions (Poston et al., 2020). Such inhibition by environmental toxins may have significant neurodevelopmental implications.

#### Calcineurin (CaN)

Calcineurin (CaN) is a phosphatase associated to L-type channels by the AKAP family of proteins. CaN is activated when it binds Ca^2+^/CaM on its catalytic subunit, and Ca^2+^ on its B subunit (Groth et al., 2003). Its affinity for Ca^2+^/CaM is much higher than the CaM-dependent protein kinases, and consequently initial elevations of intracellular Ca^2+^ preferentially induce CaN-mediated phosphatase activity over CaM kinase signaling (Groth et al., 2003). Inhibition of CaN by FK506 or cyclosporine A blocks CREB-dependent gene expression induced by depolarization and does not inhibit depolarization-induced Ca^2+^ influx or CREB Ser-133 phosphorylation (Kingsbury et al., 2007; España et al., 2010). Calcium and cAMP signaling converge on CREB-dependent gene transcription through CRTC1/TORC, a CREB transcriptional coactivator (Kovács et al., 2007). Active CaN phosphorylates CRTC1, causing it to translocate from the cytosol to the nucleus, where it accumulates and initiates expression of CREB-target genes, including *Sik1* (España et al., 2010,Li et al., 2009). SIK1 phosphorylates CRTC1, reversing translocation and depleting it from the nucleus. This negative feedback mechanism prevents persistent CREB/CRTC1-dependent transcription and dendritic growth in the face of long lasting neuronal activity (Li et al., 2009).

### Chromatin Architecture at IEGs

In addition to activating CREB and CREB co-activators like CRTC1, CBP, and p300, extracellular KCl induces changes in the chromatin architecture at IEGs (West et al., 2002; Kim et al., 2010; Pruunsild et al., 2011). Neuronal depolarization by KCl induces changes in histone H1 and H3.3, concomitant with IEG transcription (Michod et al., 2012; Azad et al., 2018). KCl also induces activity-dependent enhancers, cis-element activity, and DNA breaks regulating transcription (Pruunsild et al., 2011; Malik et al., 2014; Madabhushi et al., 2015). Promoters and enhancers of rapid IEGs –a subset of IEGs which are transcribed almost immediately after activity– are marked by paused RNA polymerase II and an accessible chromatin state (Saha et al., 2011; Tyssowski et al., 2018). Delayed IEGs –a subset of IEGs that occur post-activity later in the hour– do not have similar chromatin features at their promoters and/or enhancers. Sustained activity-dependent transcription of delayed IEGs and other secondary response genes (Tyssowski et al., 2018) may be epigenetically regulated by chromatin remodeling (Martinowich et al., 2003) and negative feedback by histone deacetylases (Kyrke-Smith and Williams, 2018). The specifics of these transcriptional regulatory mechanisms may be highly dependent on cell identity, transcriptional history, and gene or even transcript identity.

## Effects of Duration and Doses of Elevated External Potassium

Different types and durations of neuronal stimulation can induce different gene expression programs (Tyssowski et al., 2018). Moreover, the mode of Ca^2+^ entry into the neuron plays a key role in determining which signaling pathways and gene expression programs are activated (Dolmetsch et al., 2001; Martinowich et al., 2003; Kingsbury et al., 2007). While extracellular KCl concentration and membrane voltage are directly proportional (Leslie et al., 2001; Somjen, 2002), signaling strength may be non-linearly correlated with Ca^2+^ influx levels (Wheeler et al., 2008). Here, we will discuss variations in the types of extracellular KCl protocols published for *in vitro* work, including pre-treatments, variations in KCl concentration ([KCl]), and variations in time of treatment.

### Silencing Activity With Pre-Treatments

Experimentation with KCl and *in vitro* neuronal cultures began in the 1980s with treatments at 20-85 mM and treatment times between 10 minutes and 48 hours, with no pre-treatments to silence basal activity (Greenberg et al., 1985; Morgan and Curran, 1986; Bartel et al., 1989; Collins et al., 1989). The addition of Ca^2+^ channel inhibitors like APV and CNQX used in conjunction with KCl treatments distinguished the roles of NMDARs, non-NMDARs (AMPARs), and voltage-sensitive calcium channels (VSCCs) in *c-fos* and *Bdnf* induction (Zafra et al., 1990; Bading et al., 1993; Ghosh et al., 1994). Many authors have subsequently employed inhibitors in the same manner, including APV and D-AP5 (NMDARs), CNQX and NBQX (AMPARs), nimodipine and nifedipine (L-type VSCCs), KN93 (CaMK), and PD (MEK) (Dolmetsch et al., 2001; Redmond et al., 2002; Kingsbury et al., 2007; Evans et al., 2013; Cohen et al., 2018). These inhibitors are usually added to neuronal cultures several minutes (15-30 min) prior to KCl-depolarization to allay concerns about effectivity. For the experiments cited above, these inhibitors were used as experimental variants, and were compared to KCl-treatment without inhibition.

Historically, as KCl became a common means of depolarization, inhibitors became a means of standardizing activity levels and/or conferring specificity to the channels available for Ca^2+^ influx. Pre-silencing all conditions with TTX and APV excludes NMDARs from the response to depolarization. Some cultures are treated overnight in 1µM TTX to reduce endogenous activity (Xia et al., 1996; Tao et al., 1998, 2002; Lyons et al., 2012; Michod et al., 2012; Malik et al., 2014; Lyons et al., 2016). 100 µM APV is added 30 minutes prior to potassium depolarization to block the NMDARs (Tao et al., 1998, 2002). Some experiments vary concentration and time of pre-treatment, or add other inhibitors, like CNQX (Xia et al., 1996; Grubb and Burrone, 2010; Malik et al., 2014; Cohen et al., 2018; Tyssowski et al., 2018). Experimenters must decide whether pre-treatment to silence synaptic activity is appropriate for their KCl depolarization experiments. For those attempting to isolate the activity of the L-type Ca^2+^ channels, TTX + APV + CNQX pre-treatments may be useful. However, some treatments have their own constraints and effects; for instance, prolonged neuronal silencing with TTX triggers homeostatic changes (Turrigiano and Nelson, 2004), which may add artifacts to observations. Also, washout of TTX after prolonged application induces elevated activity (Saha et al., 2011; Lyons et al., 2016). Pre-silencing with TTX may be unnecessary for many experimental paradigms, especially considering the activity silencing effects of KCl concentrations above 10mM (see Figure 1).

### Variation in Stimulation by Concentration and Time

While 50-55 mM KCl has become the standard treatment concentration in the excitation-transcription coupling field, the range historically spans from low doses of 3 mM to high doses of 120 mM. According to the Goldman equation, 50 mM is sufficient to induce a +48 mV depolarization in cultures maintained in media with 5 mM KCl. Experimentally, 50mM KCl has been shown to depolarize the membrane to about -30mV (Furness, 1970; Somjen, 2002). This does not bring the resting membrane potential to zero, but it is sufficient to induce robust gene expression. We have found that lower concentrations of KCl (mild depolarization) are also potent inducer of neuronal IEG transcription (Rienecker et al., 2020). Treatment times also vary widely, from 10 seconds to 48 hours. Contrary to a common presumption, prolonged treatment with reasonable concentrations of elevated external potassium does not induce apoptosis; instead, it facilitates cell survival (see “Cell Viability Under KCl Treatment” section). Furthermore, different gene programs are triggered by different duration of KCl-induced (or, any) depolarization (Tyssowski et al., 2018). However, to our knowledge, there has not been a methodical investigation into whether varied concentrations and treatment times interact to produce different gene expression profiles. We might expect variation, as different concentrations of intracellular Ca^2+^ and modes of Ca^2+^ entry differentially induce different signaling pathways, resulting in distinct transcriptional profiles (Rajadhyaksha and Kosofsky, 2005; Tyssowski et al., 2018; Rienecker et al., 2020). In Table 1, we have compiled and sorted selected papers by KCl concentration and time of treatment.

**Table 1.** KCl Treatments by Time and Concentration.

	0–5'	6'–30'	31'–60'	1.1–2 hr	2.1–4 hr	4.1–6 hr	6.1–8 hr	8.1–12 hr	2.1–23 hr	24 hr	48 hr
3**–**8 mM								(Golbs et al., 2011)			(Leslie et al., 2001)
10 mM		(Collins et al., 1989; Evans et al., 2013; Madabhushi et al., 2015)			(Evans et al., 2013)						(Leslie et al., 2001; Evans et al., 2013; 2017)
12.5 mM		(Azad et al., 2018)	(Azad et al., 2018)				(Azad et al., 2018)				(Leslie et al., 2001)
15 mM	(Kato et al., 1998)				(Evans et al., 2017)						(Grubb and Burrone, 2010)
20–25 mM		(Collins et al., 1989; Pruunsild et al., 2011)	(Pruunsild et al., 2011)	(Collins et al., 1989; Pruunsild et al., 2011)	(Pruunsild et al., 2011)	(Pruunsild et al., 2011)	(Pruunsild et al., 2011)	(Pruunsild et al., 2011)	(Kingsbury et al., 2007)	(Collins et al., 1989; D’Mello et al., 1993)	(D’Mello et al., 1993; Bok et al., 2007)
30–35mM	(Kato et al., 1998)	(Collins et al., 1989)									
40 mM	(Collins et al., 1991)	(Collins et al., 1989; 1991)		(Cohen et al., 2018)						(Collins et al., 1989; 1991)	(Collins et al., 1989)
45 mM	(Kato et al., 1998)	(Greenberg et al., 1985; Collins et al., 1989)									
50 mM	(Kingsbury et al., 2007; Casalbore et al., 2010; Berger et al., 2018)	(Kruijer et al., 1985; Morgan and Curran, 1986; Zafra et al., 1990; Ghosh et al., 1994; Tao et al., 1998; Kingsbury et al., 2007; Li et al., 2009; Casalbore et al., 2010; Berger et al., 2018)	(Kruijer et al., 1985; Zafra et al., 1990; Ghosh et al., 1994; Tao et al., 1998; 2002; Li et al., 2009; Casalbore et al., 2010; Berger et al., 2018; Pastuzyn et al., 2018)	(Zafra et al., 1990; Tao et al., 1998; 2002; Li et al., 2009; Casalbore et al., 2010)	(Kruijer et al., 1985; Zafra et al., 1990; Ghosh et al., 1994; Tao et al., 1998; 2002; Kingsbury et al., 2007; Michod et al., 2012; Qiu et al., 2016)	(Tao et al., 1998; 2002; Kingsbury et al., 2007; Li et al., 2009)	(Zafra et al., 1990; Ghosh et al., 1994)	(Tao et al., 1998; 2002)	(Kruijer et al., 1985; Zafra et al., 1990; Redmond et al., 2002)	(Redmond et al., 2002; Martinowich et al., 2003; Li et al., 2009)	(Crowder and Freeman, 1999; Redmond et al., 2002)
55 mM	(Tyssowski et al., 2018)	(Xia et al., 1996; Madabhushi et al., 2015; Tyssowski et al., 2018)	(Xia et al., 1996; Malik et al., 2014)	(Malik et al., 2014; Lyons et al., 2016)	(Lyons et al., 2012; Malik et al., 2014)	(Lyons et al., 2012; Malik et al., 2014; Lyons et al., 2016; Tyssowski et al., 2018)		(Tyssowski et al., 2018)			
60 mM	(Kato et al., 1998; Gotoh et al., 1999; Dolmetsch et al., 2001)	(Bartel et al., 1989; Gotoh et al., 1999; Dolmetsch et al., 2001)	(Bartel et al., 1989; Dolmetsch et al., 2001)	(Bartel et al., 1989)	(Bartel et al., 1989)	(Dolmetsch et al., 2001)					
85 mM		(Collins et al., 1989)									
90 mM	(Tao-Cheng, 2018; 2018)	(Rothman, 1985)									
120 mM+	(Kato et al., 1998)	(Rothman, 1985)									

A categorization of literature using extracellular potassium treatments by duration of treatment and concentration of potassium.

## Strengths and Limitations of Extracellular KCl Treatment

### General Assessment of Extracellular KCl as an Experimental Paradigm

Extracellular KCl treatment is a relatively easy method of inducing global depolarization in vitro to study channel physiology, signaling messengers and cascades, and gene transcription. Methods involve media replacement–partially or completely– to induce depolarization, and wash out to remove the stimulus. This method is scalable, enabling experiments across a variety of KCl concentrations applied for anywhere from seconds to days and allows studies of mild graded potential changes without sophisticated equipment. Because this method induces global depolarization, it is unsuitable for distinguishing differences amongst inter-compartmental signaling events in a neuron that may exist in the brain. For example, unlike approaches such as localized glutamate uncaging, this method cannot distinguish activity-induced local events in the synapse from their dendritic, somatic, or axonal counterparts. Sustained global depolarization also cannot replicate variable stimuli on physiologically relevant timescales. Extracellular potassium is unsuitable for exploring how temporal features of action potentials are coded into specific intracellular signaling and transcriptional programs (Fields et al., 1997; Tyssowski et al., 2018). Another consideration is that experimental readouts are often population averages where cell-specific responses, which might differ based on history of activity and cell type, remain obscure. However, in nearly uniform cultures this simultaneously strengthens detection of small populational effects. As global depolarization induces calcium influx through both synapse- and soma-associated channels, multiple signaling pathways may be recruited by simple KCl treatments. Channel-specific inhibitors, gene constructs, and other tools can be used to impose specificity on KCl-induced Ca^2+^ influx and signaling (Dolmetsch et al., 2001; Redmond et al., 2002; Kingsbury et al., 2007; Evans et al., 2013; Cohen et al., 2018).

### Cell Viability Under KCl Treatment

Elevated extracellular potassium promotes cell survival in a range of neuronal cell types and species. Reports of enhanced survival include peripheral neurons from chicken dorsal root, sympathetic, and parasympathetic ganglia (Bennett and White, 1979; Wakade and Thoenen, 1984; Koike et al., 1989; Collins et al., 1991; Crowder and Freeman, 1999), the mollusk giant neuron from ganglia tissue (Kostenko et al., 1982), murine cerebellar neurons (Didier et al., 1989), and neonatal rat cortical and cerebellar neurons (Lasher and Zagon, 1972; Golbs et al., 2011). Optimal potassium concentrations that promote survival lie between 25-40 mM, with survival falling off above 50 mM, and dropping to 20-50% of maximal survival at 85 mM (Collins et al., 1989). These cell survival effects are dependent on elevated potassium and Ca^2+^ influx through DHP sensitive L-type channels. Both must be sustained to promote cell survival; calcium chelators, replacement of elevated potassium with normal media, or L-type channel inhibition blocks the protective effects (Collins et al., 1989; Koike et al., 1989; D’Mello et al., 1993). Likewise, the L-type channel agonist BAYK 8644 enhances the protective effect, achieving high survival rates at lower potassium concentrations. BAYK 8644 allows L-type channels to open at significantly lower levels of depolarization, increasing Ca^2+^ influx (Collins et al., 1989). While there is a strong quantitative correlation between mean sustained intracellular calcium levels and the percentage of surviving neurons (Collins et al., 1991), simple flux in intracellular calcium concentration is not the mechanism of neuroprotection. Concentrations of 90-200 mM KCl are reported to be lethal to glutamate sensitive neurons (Rothman, 1985; Choi, 1987; Mattson et al., 1988; Collins et al., 1989). Moreover, levels of intracellular calcium which are protective when induced by potassium-mediated depolarization, are toxic when induced by glutamate signaling, particularly in the presence of NMDARs including GluN2 (Rothman et al., 1987; Collins et al., 1989; Martel et al., 2012). This is likely because potassium-mediated depolarization relies on L-type VSCCs, whereas glutamate signals through NMDARs (Kudo and Ogura, 1986; Murphy et al., 1987; Choi, 1988; Collins et al., 1989). Ca^2+^ influx through non-NMDARs is neither sufficient nor necessary for glutamate-induced neurotoxic injury, suggesting the mode of Ca^2+^ entry determines toxicity or neuroprotection (Choi, 1988; Collins et al., 1989). Likewise, potassium-mediated and neurotrophic neuroprotection mechanisms are distinct; DHP inhibition of L-type channels does not block neuronal survival mediated by neurotrophins (Collins et al., 1989). The neuroprotective effects of elevated extracellular potassium are rather mediated by calcium-dependent intracellular signaling, although the relative importance of these different pathways may vary with neuronal identity. In neonatal rat spiral ganglion neurons treated with 25mM K^+^ for 96 hrs, nuclear CaMKIV promotes survival by activating CREB (see Figure 2), while CaMKII functionally inactivates the proapoptotic regulator Bad (Bok et al., 2007). At this sustained depolarization, CaN signaling is inactivated (Bito et al., 1997; Groth et al., 2003). The mechanism of elevated potassium-mediated neuroprotection may also partially depend on the treatment’s effect on neuronal activity. A small elevation in extracellular potassium to 8 mM reportedly shift the frequency distribution of neuronal activity toward high-frequency bursts associated with reduced caspase-3 dependent apoptosis and increased neuronal survival (Golbs et al., 2011). In cortical neurons, this neuroprotective effect of high-frequency activity is mediated by a PI3K dependent pathway (Golbs et al., 2011). The PI3K/Akt pathway is involved in both depolarization and neurotrophin promoted survival in sympathetic neurons, but may not be a universal neuroprotective mechanism in all cell types (Crowder and Freeman, 1999). At extremely high concentrations (exceeding 85mM), elevated extracellular potassium becomes antagonistic to neuronal survival due to the osmotic effects of excess sodium and chloride influx. Removal of chloride from the external medium prevents toxicity of 90mM to 140mM K^+^ (Rothman, 1985). In summary, while the optimal extracellular potassium concentration for cell survival may vary between differing neuronal types, KCl is generally neuroprotective at concentrations between 25-40 mM and at lower concentrations when supplemented with BAYK 8644. Mechanistically, this neuroprotection is mediated by Ca^2+^ dependent intracellular signaling including calmodulin kinases (CaMKII and CaMKIV) and the PI3K/Akt pathway, but the relative importance of different pathways to neuroprotection may also depend on the cell type and strength of the depolarization.

### Implications of In Vitro KCl Research for In Vivo Functions

Extracellular KCl is useful for investigating cellular mechanisms of neurons *in vitro*. However, concerns are frequently raised about its relevance to events *in vivo.* KCl treatments are indeed different from physiological stimuli in both dose and duration of application. Many protocols apply 50-55mM KCl for hours or days at a time, periods that do not reflect the duration of sensory stimuli or seizures. Nevertheless, many mechanisms mediating responses to elevated extracellular KCl also prove necessary for similar *in vivo* functions. Seizures, sensory stimuli, and learning events induce IEGs likewise induced by extracellular KCl (Morgan et al., 1987; Hunt et al., 1988; Guzowski et al., 2001; Lyons and West, 2011; West and Greenberg, 2011; Mukherjee et al., 2018; Heinz and Bloodgood, 2020). Furthermore, while sustained global depolarization by elevated KCl is not a feature of healthy neuronal signaling, it is a proposed mechanism of Leão’s spreading depression of neuronal activity (Leao, 1944; Grafstein, 1956; Somjen, 2001; de Curtis et al., 2018). Below we discuss the *in vivo* phenomena that highlight the relevance of *in vitro* findings from research using extracellular KCl.

#### IEG Transcription Is Important for Learning and Memory, and Is Induced in Physiological Conditions

IEG induction is a prominent consequence of elevated extracellular KCl treatment (Zafra et al., 1990; West et al., 2002; Tyssowski et al., 2018). While there are key differences between KCl and physiological stimuli, induction mechanisms discovered *in vitro* have held true *in vivo* (Greer and Greenberg, 2008). Transcriptional responses to depolarizing KCl *in vitro* have downstream effects on homeostatic plasticity, dendritic growth, and the remodeling of synaptic structures (Leslie et al., 2001; Redmond et al., 2002; Kovács et al., 2007; Grubb and Burrone, 2010; Tao-Cheng, 2018). Likewise, similar programs of IEG transcription *in vivo* are crucial for homeostatic plasticity, neurite outgrowth, synapse development and strength, neural adaptations to drugs of abuse, and learning and memory (Ploski et al., 2008; Lyons and West, 2011). These IEG programs are so reliable, expression of genes such as *Bdnf, Arc,* and *Fos* are often used in immunohistochemistry as proxies of neural activity induced by learning paradigms and sensory stimuli (West and Greenberg, 2011; Lyons et al., 2016).

#### L-Type Channels and Ca2+ Signaling Are Involved in IEG Transcription, Learning and Memory, and Homeostatic Plasticity

Both in culture and under physiological conditions, IEG transcription is regulated both by synaptic (NMDAR, AMPAR signaling) and extra-synaptic signaling, including somatically (L-type VSCCs) distributed ion channels. *In vitro,* extracellular KCl generates Ca^2+^ influx through L-type VSCCs and stimulates signaling to IEG transcription in the nucleus. Such signaling is highly relevant *in vivo* where L-type VSCCs induce IEG transcription and are involved in learning and memory, as well as in pathological brain functions, autism, aging, and pain (Shinnick-Gallagher et al., 2003; Jerome et al., 2012; Berger and Bartsch, 2014; Ghosh et al., 2017; Navakkode et al., 2018; Roca-Lapirot et al., 2018). Models of aging and neurodegenerative diseases like Parkinson’s disease suggest alterations in Ca^2+^ homeostasis through L-type VSCCs increase vulnerability to cognitive decline and synaptic dysfunction (Berger and Bartsch, 2014; Navakkode et al., 2018). Genome wide association studies (GWAS) and natural channel variants in humans link L-type VSCCs to working memory performance (Berger and Bartsch, 2014). Olfactory and fear memory in rats also depends on L-type VSCCs. L-type VSCCs induce transcription of the plasticity-related genes necessary for protein-synthesis dependent long term olfactory memory (Jerome et al., 2012; Ghosh et al., 2017). In brain slices from fear-conditioned rats, nimodipine reduces EPSC amplitude and increases paired pulse facilitation. When administered *in vivo* by intraperitoneal-injection, nimodipine also blocks fear-potentiated startle in a dose dependent manner (Shinnick-Gallagher et al., 2003). L-type VSCCs play a selective role in long-term, but not short term, fear conditioning memory by activating Ca^2+^/calmodulin and MAPK/ERK signaling to induce CREB-mediated transcription (Schafe et al., 2000; Bauer et al., 2002).

Extracellular KCl *in vitro* induces Ca^2+^ dependent signaling cascades involving calmodulin kinases, MAPK/ERK, and calcineurin (Ghosh and Greenberg, 1995; Rajadhyaksha and Kosofsky, 2005; Greer and Greenberg, 2008; Hagenston and Baing, 2011; Tyssowski et al., 2018). *In vivo*, elimination of CaMKII prevents activity-dependent expression of IEGs like *Bdnf, Fos,* and *Arc*, inhibits persistent synaptic strengthening, and impairs spatial memory (Cohen et al., 2018). Blockade of MAPK/ERK activation in the lateral amygdala impairs fear memory consolidation and synaptic plasticity *in vivo* (Schafe et al., 2000). CRTC1, aka TORC1, is downstream of CaN signaling and is crucial for contextual fear and spatial memory (España et al., 2010; Nonaka et al., 2014; Uchida et al., 2017). Knockdown of CRTC1 in CA1 and CA3 reduces long-term but not short-term contextual fear memory, implying its effect on activity-induced gene transcription, which is necessary for long-term memory. In response to neural activity and learning, CRTC1 localizes to the nucleus in a CaN dependent manner and induces *Fgf1b*. (Uchida et al., 2017). Furthermore, gene transcription mediated by CRTC1, including *fos, Bdnf,* and *Nr4a2*, is impaired in Alzheimer’s disease transgenic mice expressing human beta-amyloid precursor protein. These transcriptional deficits coincide with long-term spatial memory deficits in the transgenic mice (España et al., 2010).

Homeostatic alterations and remodeling of cellular structures are also consequences of global depolarization by KCl (Leslie et al., 2001; Grubb and Burrone, 2010; Evans et al., 2013; Tao-Cheng, 2018, 2018). In hippocampal cultures, chronic depolarization with high potassium moves the axon initial segment (AIS) away from the soma, dependent on the activation of L-type VSCCs (Grubb and Burrone, 2010). Changes in AIS position and activity-dependent plasticity are thought to fine tune neuronal excitability according to ongoing electrical activity (Grubb and Burrone, 2010). In *Aplysia,* subthreshold depolarizing changes in the presynaptic holding potential of the sensory neuron B21 increase the rate at which homosynaptic facilitation occurs. L-type VSCC-dependent calcium elevation is partially responsible for these membrane potential-induced alterations in facilitation (Ludwar et al., 2020).

#### Potassium Hypotheses of Brain Disorders

Brain disorders such as migraine aura, epilepsy, haemorrhagic and ischaemic stroke, subarachnoid hemmorahage, and traumatic brain injury are associated with a robust phenomenon called spreading depression (SD), first described by Leão in 1944 and subsequently linked to increases in extracellular K^+^ *in vivo* (Leao, 1944; Somjen, 2001; Gupta, 2006; Fabricius et al., 2008; Richter and Lehmenkühler, 2008; Charles and Brennan, 2009; Cui et al., 2014; de Curtis et al., 2018). SD is a transient (60-120s), self-propagating wave of neuronal and glial depolarization accompanied by a negative shift of the current potential of about 20-35 mV and followed by prolonged quiescence (Richter and Lehmenkühler, 2008; Zhang et al., 2011; Cui et al., 2014). This propagation occurs in all directions at ∼3 mm/min, a rate similar to the spread of aura in classical migraine (Richter and Lehmenkühler, 2008; Cui et al., 2014). SD is accompanied by changes in blood flow, vascular caliber, and energy metabolism. While SD can occur in all neural tissues (Charles and Brennan, 2009; Dong et al., 2020), it is most often studied in the cortex, where it is referred to as cortical spreading depression (CSD). The phenomenon occurs in rat, mouse, rabbit, pigeon, and human models, among others (Leao, 1944; Fabricius et al., 2008; Richter and Lehmenkühler, 2008; Charles and Brennan, 2009).

The mechanistic hypothesis of SD postulates increases in extracellular [K^+^] trigger massive neuronal depolarization that leads to network hyperactivity and further K^+^ accumulation, which eventually depresses activity (Grafstein, 1956; Somjen, 2001; de Curtis et al., 2018). When the brain tissue is unable to clear the excessive K^+^ in the extracellular space, prolonged accumulation of K^+^ and sustained depolarization compromises synaptic transmission (de Curtis et al., 2018). Mild depolarization moves the membrane potential closer to the firing threshold and increases excitability, but stronger depolarization (as extracellular [K^+^] continues to rise) inactivates some voltage sensitive ion channels, raises the threshold, and eventually–reminiscent of *in vitro* findings mentioned in “Elevated Extracellular KCl Induces Neuronal Depolarization, But Often, Not Activity” section–suppresses excitability (Kager et al., 2002; Somjen, 2002; Dreier et al., 2012). During SD, increases in extracellular [K^+^] above the baseline 3 mM have been reported, ranging from 7.3 mM (Enger et al., 2015) up to 35 mM (Kraio and Nicholson, 1978), and are accompanied by a sharp drop in extracellular Na^+^ and Ca^2+^. This suggests K^+^ leaving the cells is exchanged against an influx of Na^+^ and Ca^2+^ (Brinley et al., 1960; Nicholson, 1984; Silver and Erecńska, 1990; Somjen, 2001). Membrane potentials consequently depolarize significantly, briefly approaching zero (Somjen, 2001). In K^+^ induced models of CSD *in vivo*, K^+^ rise precedes CSD and intracellular increases in Ca^2+^. These neuronal Ca^2+^ transients lag behind the negative direct current potential shift but last twice as long. Interestingly, astrocytic Ca^2+^ increases lagged further behind negative potential shifts, and were shorter than neuronal Ca^2+^ transients (Enger et al., 2015). Astrocytes can prevent the occurrence of SD through buffering of extracellular K^+^ (Cui et al., 2014). However, glial cells also experience the depolarization induced by SD, and extracellular [K^+^] may exceed their capacity to maintain physiologically tolerable limits. As neuronal dendrites and glia uptake K^+^ and swell, they restrict the interstitial space and further concentrate extracellular [K^+^] (Somjen, 2001).

Both elevations in extracellular [K^+^] and glutamate contribute to SD, but synaptic signaling is not required (Brinley et al., 1960; Charles and Brennan, 2009; Enger et al., 2015). CSD becomes less frequent and harder to trigger if P/Q calcium channels are genetically modified or specifically blocked. Likewise, blocking NMDARs by MK-801 stops the spread of CSD (Richter and Lehmenkühler, 2008). TTX, however, does not block the propagation of CSD, even though it suppresses action potential firing. Most likely, increases in extracellular [K^+^] and neuronal voltage-activated currents are key to the ignition and evolution of SD, as opposed to interstitial glutamate (Enger et al., 2015). However, extracellular [K^+^] is reported not to increase ahead of changes in voltage, as it should if it were the agent of propagation of SD (Somjen, 2001).

The difference between *in vitro* KCl treatments and *in vivo* SD is that SD events accompany other activity. In epilepsy for instance, SD interacts with synchronous ictal epileptic events and paroxysmal depolarization shifts (Dreier et al., 2012; Kubista et al., 2019; Zakharov et al., 2019). Thus, it is ambiguous whether the *in vivo* cellular consequences of SD associated with epilepsy relate to increased synchronous activity, or to the sustained depolarization that follows. Furthermore, as SD propagates across neural tissue at ∼3 mm/min, extracellular [K^+^] rises more slowly in physiological conditions than KCl treatments applied *in vitro*. The rate of depolarization by extracellular [K^+^] may also impact membrane voltage and cellular responses. Nevertheless, extracellular KCl can be applied to induce SD in experimental models *in vivo* (Marshall, 1959; Jing et al., 1991; Herdegen et al., 1993; Herreras and Somjen, 1993; Busch et al., 1996; Fujikawa et al., 1996; Somjen, 2001; Cui et al., 2014). While comparisons between *in vitro* KCl treatments and physiological events like SD must be cautious, they could be reciprocally informative.

### Potential Use of Extracellular KCl in Other Fields

Fields broader than that of activity-regulated transcription may benefit from using potassium-mediated depolarization *in vitro*. For example, in environmental toxicology, toxins like polybrominated diphenyl ethers (PBDEs) and methylmercury (MeHg) act via pathways involving neuronal excitability, intracellular calcium concentrations, and MAPK, PKA/CREB, and PI3K/Akt signaling. PBDEs are a bioaccumulative class of brominated flame retardants with neurodevelopmental toxicity. PBDE metabolites like 6-OH-BDE-47 impair MAPK signaling (Poston et al., 2020) and disturb intracellular [Ca^2+^] by causing an influx of extracellular Ca^2+^ and release of Ca^2+^ from intracellular stores in the ER and mitochondria (Dingemans et al., 2010). Disruption of normal MAPK signaling to IEGs and of excitability could play a role in the neurodevelopmental toxicity of PBDEs. Likewise, methylmercury increases intracellular Ca^2+^, disrupts MAPK and ERK1/2 signaling, and upregulates IEGs like fos, c-jun, and Bdnf (Bulleit and Cui, 1998; Dao et al., 2017; Fujimura and Usuki, 2017; Heimfarth et al., 2018, 2018). MeHg induced MAPK and PKA/CREB pathways trigger site specific neural hyperactivity and degeneration (Fujimura and Usuki, 2017, 2018; Heimfarth et al., 2018). These similarities with mechanisms of potassium-mediated depolarization may prove informative.

### Wrap Up

Elevated extracellular potassium is a useful *in vitro* treatment for elucidating the signaling, transcriptional, and plasticity-related events associated with global neuronal depolarization. Because it is applied by media replacement, it is scalable by dose and time. Many *in vitro* depolarization-induced mechanisms, including Ca^2+^ influx through L-type VSCCs, Ca^2+^ dependent signaling, IEG transcription, homeostasis, and long term potentiation, are also recruited for sensory stimuli, learning, memory, disease, and seizures *in vivo*. Indeed, KCl treatments have strong similarities to the potassium hypothesis of SD and epilepsy. However, researchers should justify the parameters of KCl treatments cautiously; prolonged periods, rapid change in concentration, and high doses of depolarizing KCl are not comparable to *in vivo* stimuli. Given the wide range of potassium stimulation protocols, more investigation is required into whether different stimulation parameters have differing results. For example, different neuronal activity patterns (short and prolonged) induce different IEG expression profiles (Greer and Greenberg, 2008; Tyssowski et al., 2018). Consequently, treatment length, cell type, and activity history of the neurons may heavily impact findings. The selected stimulation paradigm also has consequences for the final data type. Electrophysiological recordings allow internal measurements of the depolarization of single cells and can stimulate single cells to depolarize at graded stages. However, the modulation of only one or a few cells at a time is incompatible with certain molecular biology techniques that require higher cell loads, like chromatin immunoprecipitation. KCl treatment, which can be applied homogenously to a population of cells *in vitro*, is more appropriate for these measures. While global depolarization induced by potassium recruits multiple ion channels and several signaling cascades, experimenters can still achieve some degree of specificity. Inhibitors and genetic engineering can limit the effects of global depolarization to the mechanisms of interest. Inhibitors may be used as acute treatments or prolonged pre-treatments, depending on the intended comparisons. The body of work using elevated extracellular potassium *in vitro* has significantly contributed to our understanding of depolarization-transcription coupling mechanisms in neurons, and these findings are robustly confirmed by *in vivo* work. Consolidating an understanding of the boundaries of this stimulation protocol will ensure it remains a productive research tool.

## Summary Statement

Elevated extracellular potassium chloride is widely used to achieve membrane depolarization, but there is great variability between protocols. We summarize the mechanisms of potassium-mediated depolarization, their relevance to brain functions and dysfunctions, and strengths and drawbacks of this technique.
